# Hidradenite suppurative et psoriasis: anguille sous roche

**DOI:** 10.11604/pamj.2017.27.200.3367

**Published:** 2017-07-14

**Authors:** Mouna Bouaddi, Badredine Hassam

**Affiliations:** 1 Service de Dermatologie et de Vénéréologie, Faculté de Médecine et de Pharmacie Mohammed V Souissi, Rabat, Maroc

**Keywords:** Hidradénite suppurative, psoriasis, maladies inflammatoires chroniques, VIH, Hidradenitis suppurativa, psoriasis, chronic inflammatory diseases, HIV

## Image en médecine

L'hidradénite suppurative et le psoriasis sont des maladies inflammatoires chroniques. La première siège préférentiellement au niveau des zones riches en glande apocrine, la deuxième est ubiquitaire. L'association des deux pathologies n'a jamais été décrite dans la littérature. Cette association cachait une infection à HIV évolutive. Mr MB, âgé de 45 ans, tabagique chronique depuis 10ans, était hospitalisé pour la prise en charge de nodules durs douloureux érythémateux fistulisés des régions axillaires et inguinales apparus 6 mois avant avec des lésions érythémato-squameuses des plantes (A). Les nodules ont augmenté de taille et ont conflué en placard avec apparition secondaire de fistules laissant sourdre de pus jaune nauséabond en grande abondance (B). L'état général était conservé et l'examen somatique ne relevait pas d'anomalie. Le patient a bénéficié de deux biopsies une au niveau axillaire et l'autre au niveau de la plante confirmant les diagnostics de maladie de Verneuil sur la première et de psoriasis sur la deuxième. Une sérologie HIV a été demandée, fortement positive ce qui a été confirmé par le western blot avec taux CD4>500/mm^3^. Les présentations atypiques des dermatoses et les associations inhabituelles des dermatoses ayant des mécanismes physiopathologiques très différents doivent faire suspecter une infection rétrovirale. L'association de psoriasis et de l'hidradenite suppurative n'a jamais été décrite même au cours de l'infection HIV. L'infection VIH peut occasionner une multitude de dermatoses de présentation atypique. Le dermatologue doit être vigilant pour permettre d'établir un diagnostic précoce de l'infection.

**Figure 1:**
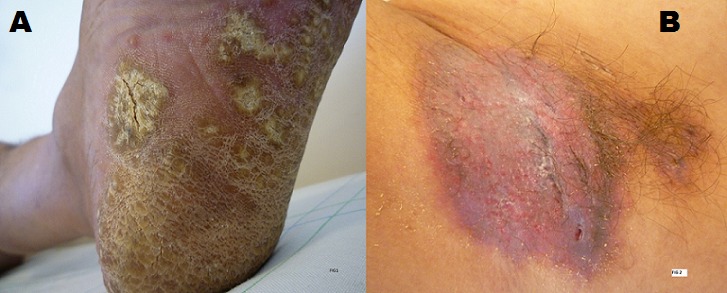
A): kératodermie plantaire; B): placard érythémateux, nodulaire et fistuleux au niveau axillaire droit

